# Wandering projectile, a rare cause of acute urinary retention

**DOI:** 10.1186/s12894-023-01204-x

**Published:** 2023-03-10

**Authors:** Yohannis Derbew Molla, Desyibelew Chanie Mekonnen, Tewodros Baye Gelaw, Tewodros Ayalew Sendekie

**Affiliations:** Department of Surgery, University of Gondar Specialized Hospital, Gondar, Ethiopia

**Keywords:** Obstruction, Retention, Gunshot wound, Urethra, Wandering projectile, Case report

## Abstract

**Background:**

Urethral obstruction due to retained projectile migrating into the genitourinary system has rarely been reported. The literature describes two main methods of retained projectile removal from the genitourinary system: (1) spontaneous expulsion during voiding and (2) manual extraction due to urethral obstruction causing acute urinary retention.

**Clinical presentation:**

We present a case in which a 23-year-old man presented with acute urinary retention four days after suffering a gunshot wound to the right distal posterolateral thigh. A retained projectile eroded through the posterior wall (slightly to the right) of the bulbar urethra at the bulb, migrated through the urethra, and eventually became lodged in the external urethral meatus, causing obstruction and acute urinary retention. Subsequently, the foreign body was removed with manual extraction along with gentle external pressure under sedation and the patient was discharged with a 16 Fr transurethral catheter in situ to be kept for 1 week and removed after a week.

**Conclusion:**

The absences of signs do not always effectively rule out urethral or bladder injury. Urethral foreign bodies are not commonly encountered when they do the entry is usually the urethral meatus. However, the treating physician must that other mechanisms also exist especially in those with bullet injury to flank, abdomen, pelvis and even the distal thigh like our case.

**Supplementary Information:**

The online version contains supplementary material available at 10.1186/s12894-023-01204-x.

## Background

Urethral foreign bodies are a relatively rare occurrence, with few case reports in the literature to describe presentation and management [1–6]. Several motivations for insertion have been described, including psychiatric illness, autoeroticism, intoxication, and perceived contraception, and other are the result of traumatic events such as war injuries, surgery or assaults, just like our case [1]. There are few case reports of projectile migration from the proximal thigh wounds eroding through the urinary bladder to the urethra. However, as far as our knowledge is concerned distal thigh projectile eroding through the urethra and migrating to the meatus is not documented in the literatures. Here we report a case that suffered a gunshot to the distal thigh. The injury site was on the right postero-lateral thigh. Few days later, the patient presented to the emergency department with a complaint of acute urinary retention due to lodged projectile at the distal urethral meatus.

## Case presentation

A 23 year old male patient presented with a complaint of failure to pass urine for the past 12 h. He had bullet injury 4 days prior to his presentation. The bullet entered on the right posterolateral distal thigh but has no exit. Associated with the trauma he has moderate bleeding from the entry site which was stopped by applying local pressure. Otherwise the patient had no any urinary complaint until 12 h prior to his presentation. The patient did not have any imaging following the trauma prior to his presentation since he sustained the trauma in rural area where imaging studies were not available. He has no history previous trauma, diabetes, hypertension or known allergy. The patient has no history of any psychiatric illness and he denied self-infliction.

**Fig. 1:**
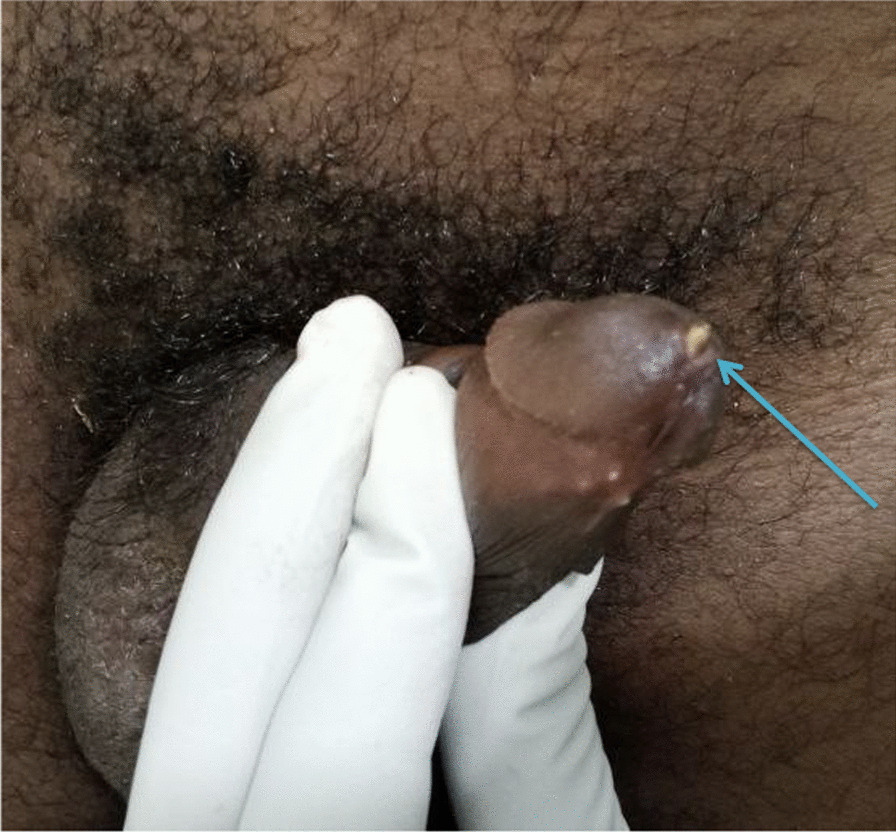
Bullet visible at the tip of urethral meatus

On examination, he was acutely sick looking and was anxious with a pulse rate of 100 bpm and blood pressure of 110/70 mmHg. Local examination revealed a distended bladder up to the umbilicus, normal male type external genitalia with uncircumcised penis. There was a palpable foreign body (projectile) at the distal penile urethra and the tip was visible at the urethral meatus (Fig. 1). The distal neurovascular structures were intact.

**Fig. 2:**
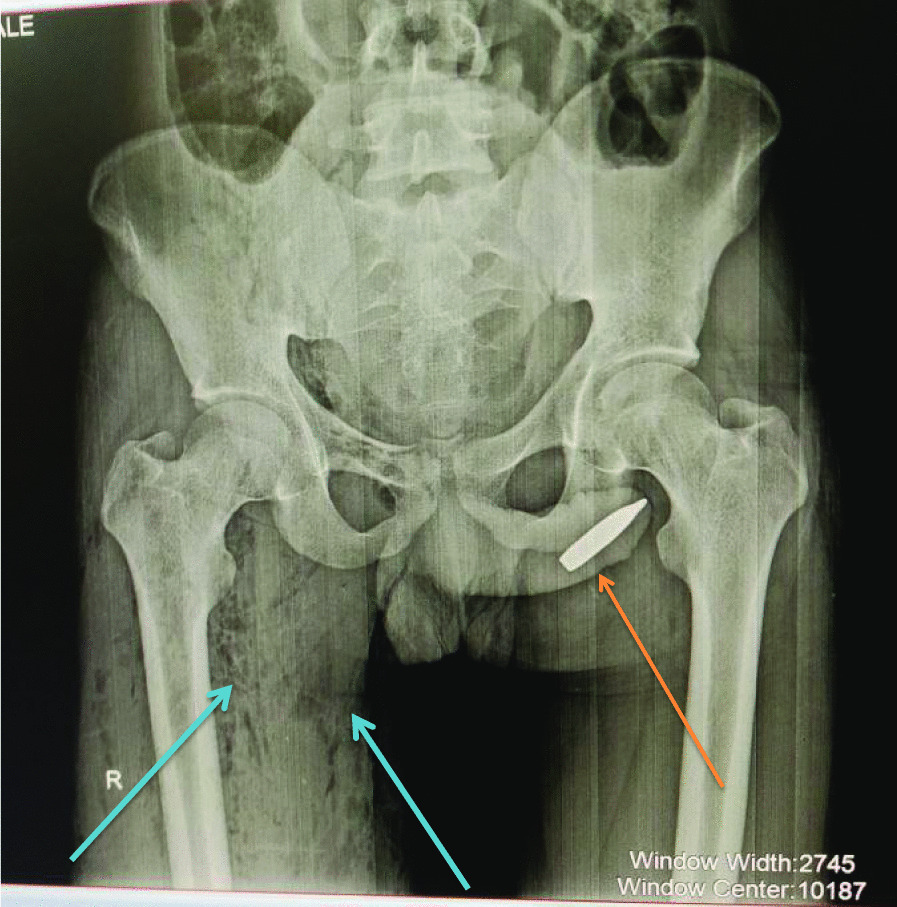
Bullet at the distal urethra and gas shadow trickling (blue arrow)

On investigations, his blood reports were within the normal range and an X-ray of the pelvis and external genitalia showed a long radio-opaque structure at the distal anterior urethra and a gas shadow trickling towards the bulbar urethra along its trajectory (Fig. 2). Computerized tomography (CT) scan was not done because our CT scan was not functional at that time.

With a diagnosis of acute urinary retention due to impacted projectile foreign body at the distal urethra, the patient was taken to the operation room. Under sedation and supine position, the foreign body was removed using artery forceps by gentle traction with gentle external pressure by a surgical assistant. After removal the patient was able to urinate by himself with no difficulty. The bullet entry site debrided and irrigated and the bullet trajectory was towards the perineum traversing the hamstring muscle but had no exit wound. Subsequently the patient was commenced on diclofenac 50 mg IV twice a day and wound care daily for the bullet entry site. The bullet entry site closed after 5 days of wound care and healed well.

**Fig. 3:**
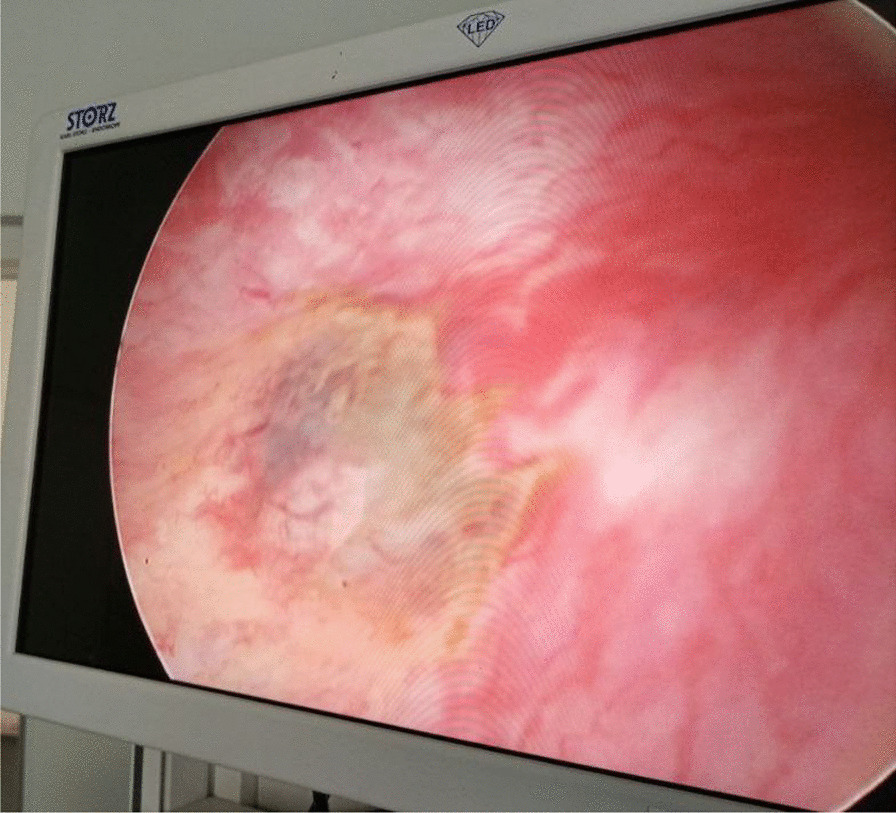
Mucosal injury at the posterior wall of bulbar urethra on cystoscopy examination

Subsequent diagnostic cystoscopy showed mucosal injury at the posterior wall (slightly to the right) of bulbar urethra with no bleeding (Fig. 3). Therefore, a 16 Fr Foley catheter was introduced per urethra and was kept in situ for 7 days and the catheter was removed after 1 week and the patient discharged improved.

## Discussion and conclusions

Urethral foreign bodies are a relatively rare occurrence. Such foreign bodies are introduced for sexual gratification and/or due to psychiatric illness. The usual symptoms of intra-urethral foreign bodies include; frequency, dysuria, hematuria, difficulty of urination and complete urinary obstruction with retention [2]. However, patients may not have urine extravasation because of elastic property of the lower urinary tract and associated edema resulting in early spontaneous closure [6].

Migration of bullet into the genitourinary tract to the urethral meatus is an unusual finding, and is especially rare when it causes acute urinary retention. Although a tremendous variety of foreign bodies have been found in the urethra, majority of these foreign bodies are introduced through the urethra. Other cases are the result of traumatic events such war injuries, surgery or assaults like our case [1].

Diagnosis of a foreign body in the urethra as a rule is not difficult. Since every part of the urethra, with the exception of the prostatic urethra, is available for palpation if one includes rectal examination, it would usually be possible to detect the object unless it were very small [3]. A plain X-ray of the pelvis is sufficient in many cases to identify these foreign bodies. However, if the foreign body is radiolucent, then other modalities such are cystoscopy and CT scan are required to confirm the presence and location [4].

Here we report a rare case of acute urinary retention due to impacted projectile at the distal urethra meatus after a gunshot wound to the right postero-lateral distal thigh. After the trauma the patient has no signs of genitourinary trauma such as bleeding per meatus or hematuria (gross hematuria is associated with 67–95% of bladder injuries) [7] for 3 days. The question as to how the bullet entered this patient’s genitourinary system remains puzzling. However, with the above cystoscopy finding and the trajectory of the bullet we assume that the bullet entered at the posterior wall of the bulbar urethra slightly to the right side which coincides with the x-ray and physical examination findings.

In one case report, a retained bullet eroded through the bladder wall, migrated through the bladder and urethra and eventually lodged into the distal urethra causing acute urinary retention after three years of bullet injury. Later the patient was managed with manual removal of the bullet with no evidence of lead on the bladder or urethra upon voiding cystourethrographic evaluation [8]. In another case report a 30 mm caliber bullet migrated from the prostatic urethra in to the anterior urethra with subsequent impaction and urinary retention after three years of the original entry of the bullet [3].

With respect to treatment of patients with urethral foreign bodies, those patients presenting with acute urinary retention like our patient require retrieval of the bullet via cystoscopic or open means. However, those patients who spontaneously expelled projectiles required no further surgical intervention for this indication [5]. Wandering projectiles causing acute urinary retention is a very rare condition and only a few case reports have been published in the literatures and unlike the previously reported cases our patient had a relatively short time between the injury and the presentation. We call it wandering because our patient had not had any urinary complaint until 12 h prior to his presentation. As far as our knowledge is concerned no case reports have been published in Ethiopia so far.

A very unusual case of foreign body in the urethra is presented with x-rays which show the migration of a large caliber bullet from the bulbar urethra into the penile urethra with subsequent impaction at the urethral meatus and caused acute urinary retention. Migration of the projectile from the bulbar urethra into the urethra meatus occurred more than three days after the original entry of the bullet to the distal thigh. The absences of signs such as hematuria, or grossly visible wounds do not always effectively rule out urethral or bladder injury and a high index of suspicion may be necessary to make the diagnosis.

## Supplementary Information

**Additional file 1**. All the pictures used in the manuscript and additional pictures were included in the supplementary file.

## Data Availability

The datasets used and/or analysed during the current study available from the corresponding author on reasonable request and all data generated or analysed during this study are included in this published article [and its Additional file 1] as well.

## Abbreviations

CT: Computer tomography
Fr: French
IV: Intravenous
