# Supplementary figures and images for: Wandering projectile, a rare cause of acute urinary retention
Source: BMC Urol. 2023 Mar 10;23:36. doi: 10.1186/s12894-023-01204-x (PMC9999539; doi:10.1186/s12894-023-01204-x)

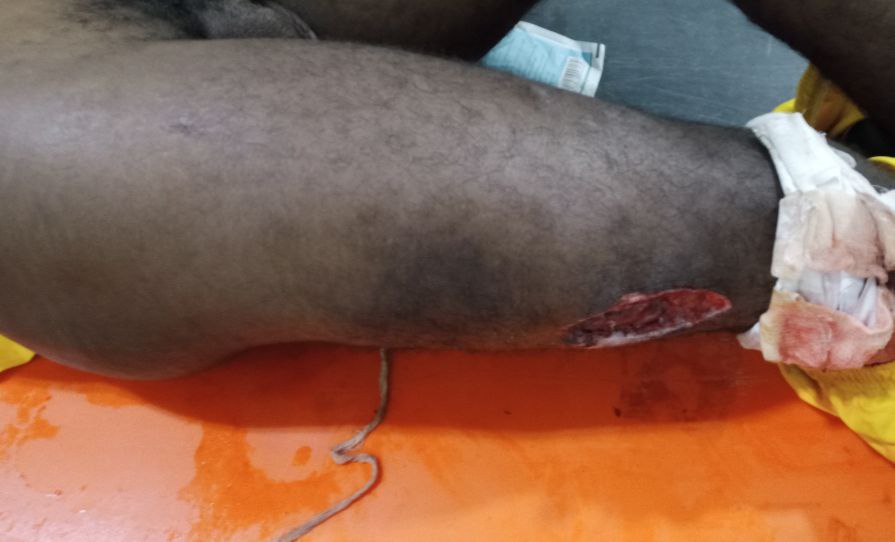


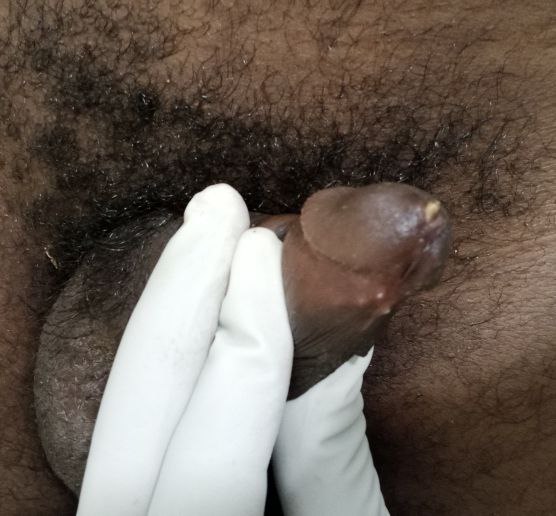


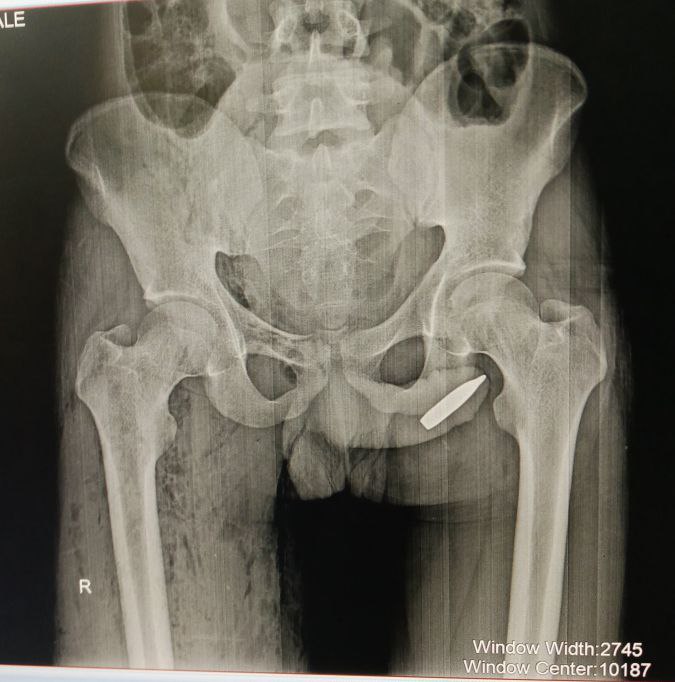


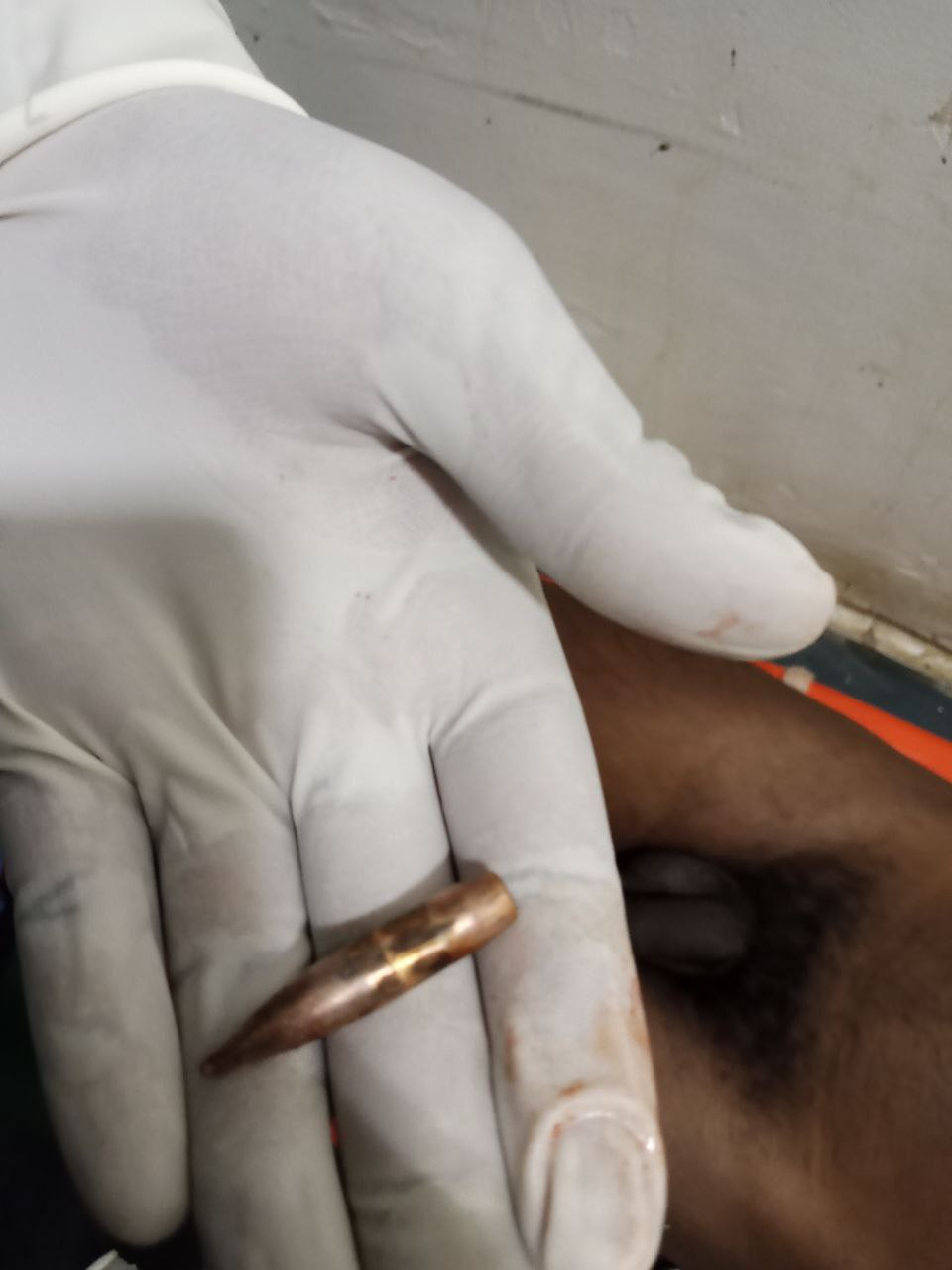


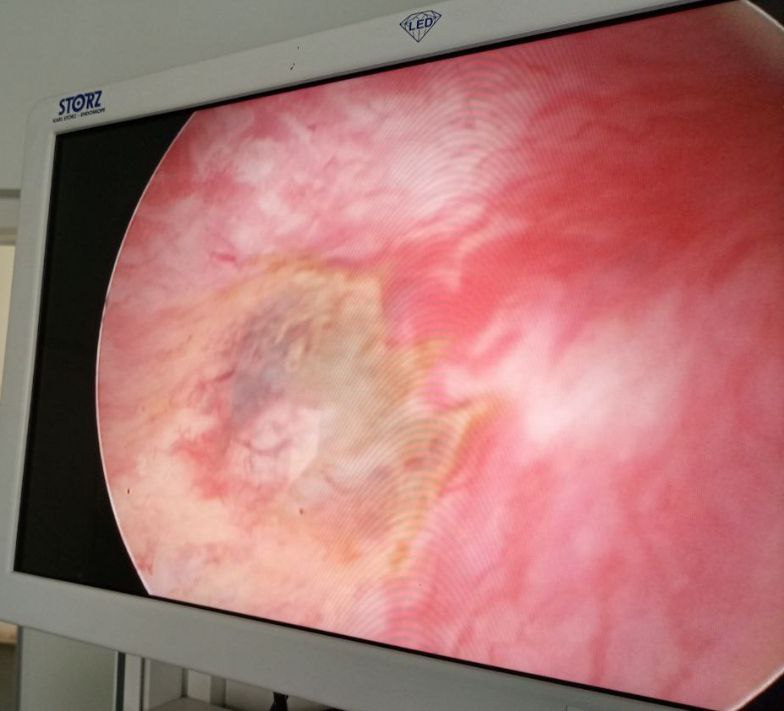


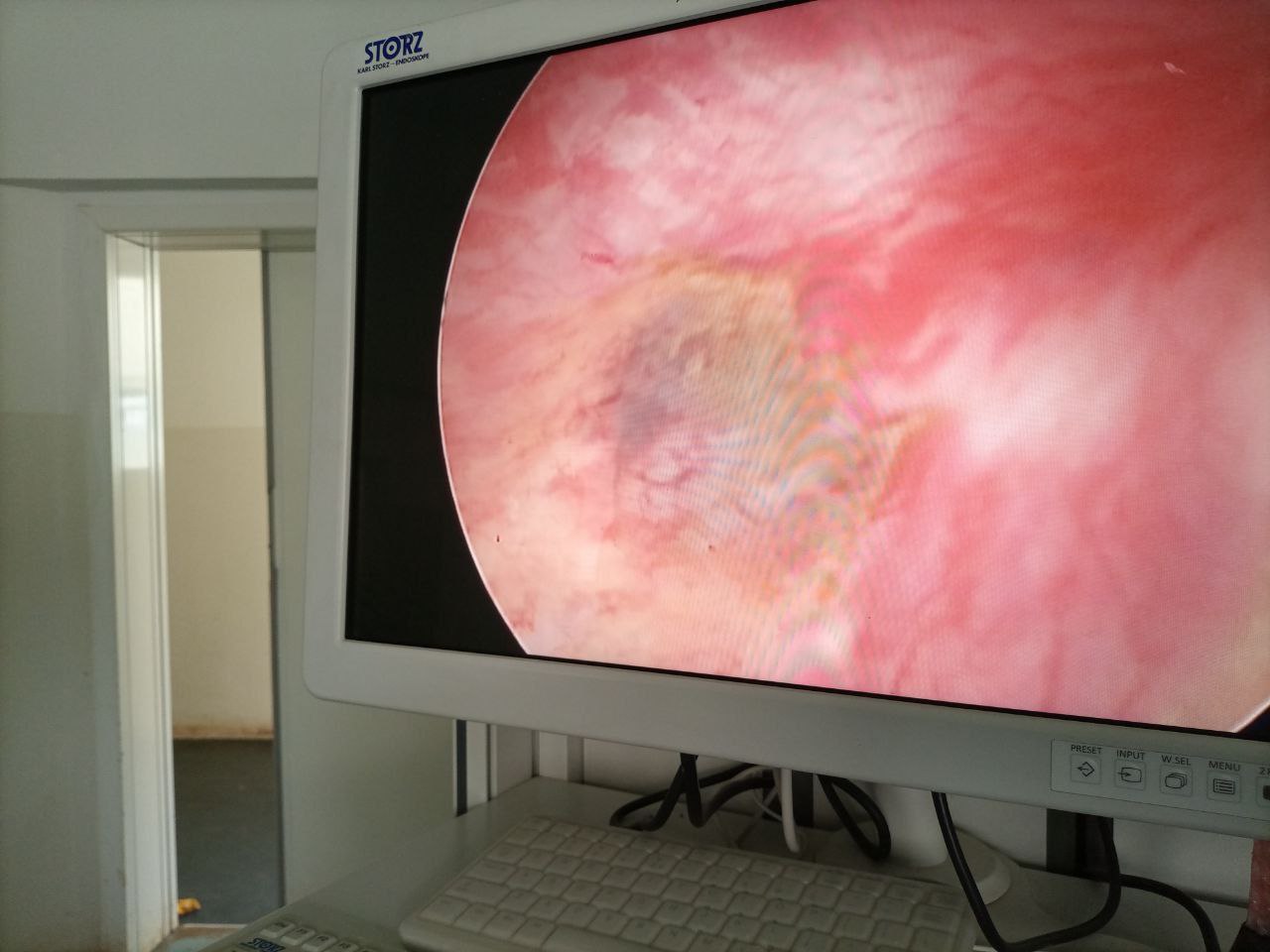

Supplement: Supplementary file 1 — Additional file 1. All the pictures used in the manuscript and additional pictures were included in the supplementary file. [file 12894_2023_1204_MOESM1_ESM.docx]
